# Supplementary figures and images for: YebC2 resolves ribosome stalling and increases fitness of cells lacking EF-P and the ABCF ATPase YfmR
Source: PLoS Genet. 2025 Apr 11;21(4):e1011633. doi: 10.1371/journal.pgen.1011633 (PMC11990639; doi:10.1371/journal.pgen.1011633)

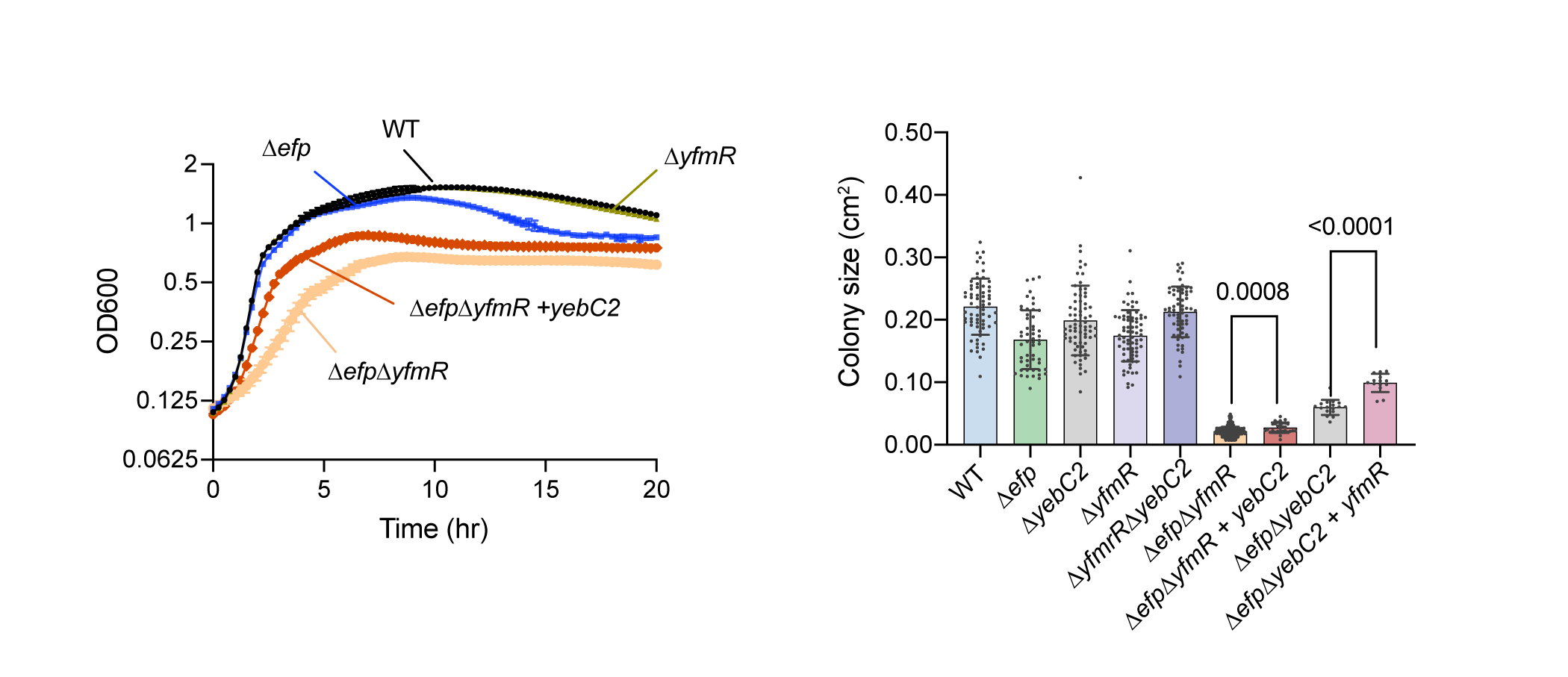

Supplement: S1 Fig — (Left) Growth in LB liquid media at 37˚C of wild-type (WT), ∆efp, ∆yfmR, ∆efp∆yfmR and ∆efp∆yfmR cells expressing IPTG-inducible YebC2. (Right) Colony sizes on LB plates of various mutants after 24 hours of growth at 37˚C. YebC2 or YfmR was expressed from an IPTG-inducible promoter. Error bars represent standard deviation. P-vaules report the result of an unpaired t-test with Welch’s correction. (TIF) [file pgen.1011633.s001.tif]

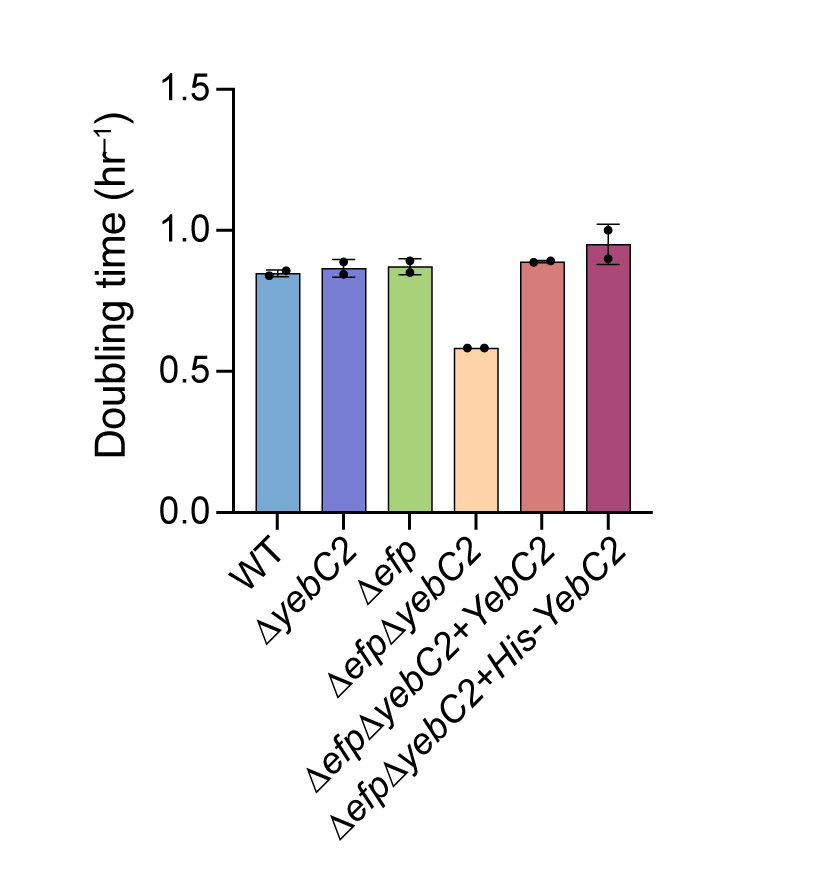

Supplement: S2 Fig — Growth rates in LB at 37˚C are shown for wild-type, ∆yebC2, ∆efp, ∆efp∆yebC2 and ∆efp∆yebC2 expressing His-tagged YebC2. Error bars represent standard deviation of two independent experiments. (TIF) [file pgen.1011633.s002.tif]

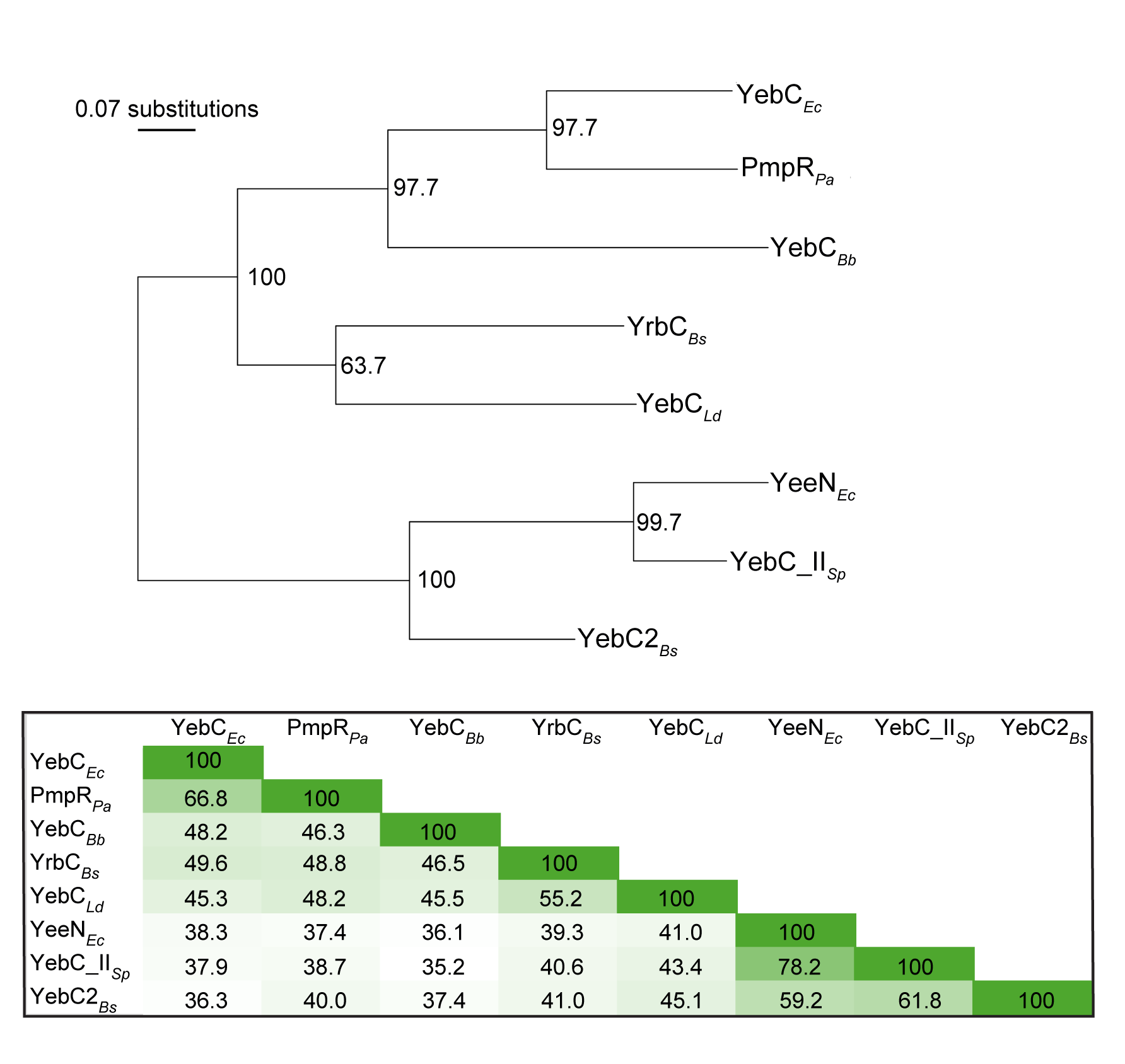

Supplement: S3 Fig — Characterized YebC family proteins are labelled with their given gene name and respective organism: Bs, Bacillus subtilis; Ld, Lactobacillus delbrueckii; Pa, Pseudomonas aeruginosa; Ec, Escherichia coli; Bb, Borrelia burgdorferi; Sp, Streptococcus pyogenes. Maximum likelihood bootstrap values are listed at each node. Pairwise percent identities for the proteins are listed and shaded relative to their homology. (TIF) [file pgen.1011633.s003.tif]

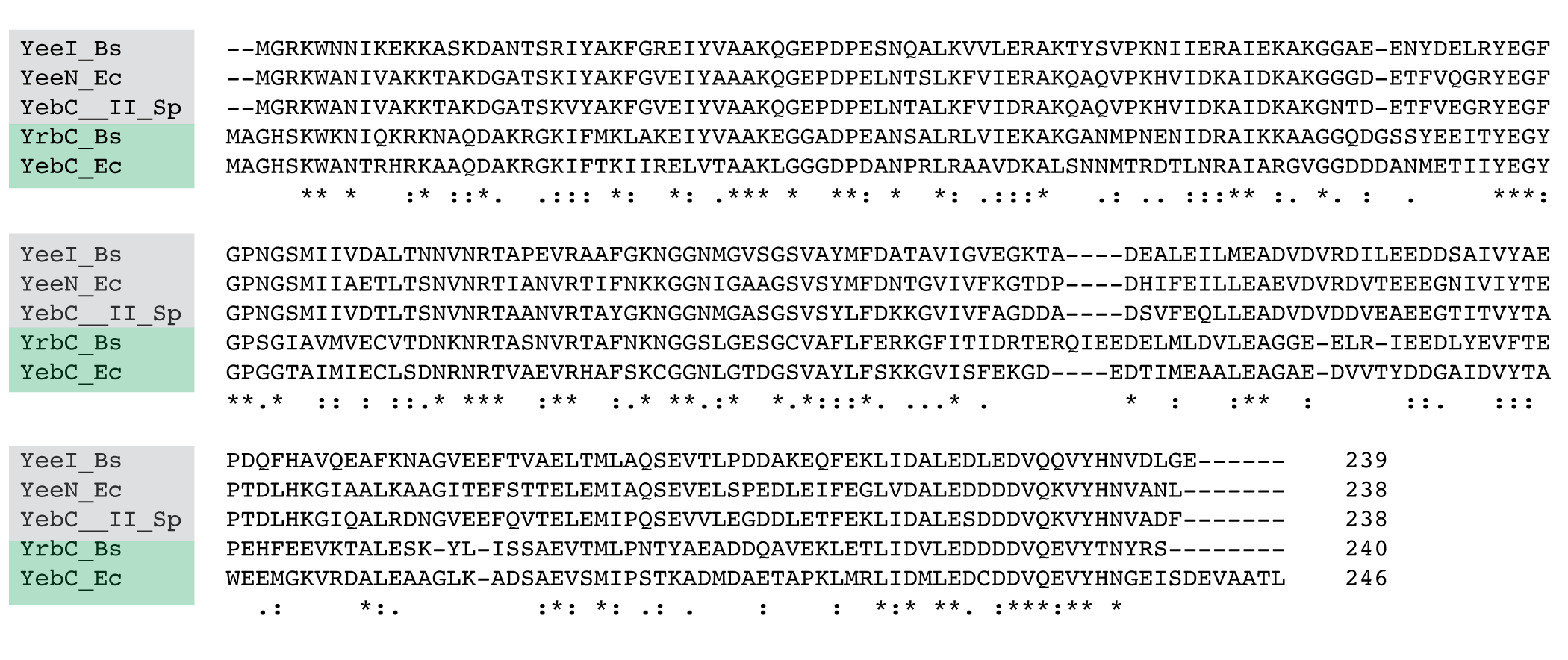

Supplement: S4 Fig — Amino acid sequences were aligned with Clustal Omega. YebC2 paralogs are shaded in gray and YebC paralogs are shaded in green. Species abbreviations: Bs, Bacillus subtilis; Ec, Escherichia coli; Sp, Streptococcus pyogenes. (TIF) [file pgen.1011633.s004.tif]
