# Supplementary material for: YebC2 resolves ribosome stalling and increases fitness of cells lacking EF-P and the ABCF ATPase YfmR
Source: PLoS Genet. 2025 Apr 11;21(4):e1011633. doi: 10.1371/journal.pgen.1011633 (PMC11990639; doi:10.1371/journal.pgen.1011633)
Supplement: Attachment — Submitted filename: Response to reviewers.pdf [file pgen.1011633.s008.pdf]

Additional Editor Comments :

While there was appreciation for the interesting data presented, more robust support for some of the conclusions stated, such as those regarding the independent action of YebC2 from EF-P and YfmR, the impact of YebC2 association with 70S polysomes, and differences between YebC and YebC2, are needed to provide the improved mechanistic detail required for PLoS Genetics.

We thank the editor and reviewers for their helpful suggestions and insight which have greatly strengthened our manuscript. We have added new data which support the ability of YebC2 to prevent ribosome stalling independent of EF-P and YfmR. We also more thoroughly determine the differences between YebC2 and its YebC paralog in *B. subtilis*. By exchanging domains between YebC2 and its paralog, we identify Domain I as being necessary for YebC2's function *in vivo*. These additional mechanistic details reveal a more complete picture of the newly identified translation factor YebC2.

Experimental Additions:

1. We have added additional data to show that YebC2 can prevent ribosome stalling independent of YfmR and EF-P. Previously, we showed that over-expression of YebC2 rescues the growth of Δ*efp*Δ*yfmR* double deletion and that loss of all three anti-stalling factors (EF-P, YfmR and YebC2) causes a more severe fitness defect than any double deletion. In the revised version, we add data with the proline stalling reporter showing that YebC2 reduces ribosome stalling at the penta-proline tract in Δ*efp*Δ*yfmR* cells. These new data are available in **Figure 3A**.
2. We have more thoroughly investigated YrbC, the YebC-like paralog of YebC2 to determine functional differences between these two proteins. We found that while deletion of *yebC2* increases ribosome stalling at a penta-proline tract in vivo, there was no measurable increase in stalling in Δ*yrbC* cells. Moreover, Δ*efp*Δ*yrbC* cells did not exhibit increased ribosome stalling in contrast to Δ*efp*Δ*yebC2* cells. Finally, expression of YrbC from an IPTG inducible promoter did not decrease ribosome stalling in this background, in contrast to expression of YebC2 from the same promoter. These data are shown in **Figure 4B**.
3. Using AlphaFold modeling, we show that while YebC2 and its paralog YrbC (YebC) are likely structurally similar, there are important differences in electrostatic charge at the surface of these proteins. In particular, in Domain I, there is a region of residues that are negatively charged in YebC2 but which are positively charged in YrbC. This modeling is shown in **Figure 4A and in Figure 5A and B**.
4. We identify Domain I as being important for YebC2's ability to prevent ribosome stalling. When we replaced YebC2's Domain I with Domain I from YrbC the chimeric protein did not complement the ribosome stalling phenotype of the Δ*efp*Δ*yebC2* double deletion. In contrast, when we replaced YrbC Domain I with Domain I from YebC2, this resulted in a functional protein that complemented the ribosome stalling phenotype of Δ*efp*Δ*yebC2*. These data suggest that residues in Domain I are functionally important and are presented in **Figure 5D**.
5. We show additional data in a more standard liquid media assay that at both 30˚C and 37˚C that YebC2 rescues the Δ*efp*Δ*yfmR* fitness defect. The rescue in liquid media at

37˚C is more pronounced than the colony-sizing data. These data are available in **Figure 2A** and in the supplementary **Figure S1**.
6. We add a Δ*efp* control strain to the experiment where we use CRISPRi to deplete efp in various mutant backgrounds in **Fig 1D**.

Text and other modifications:

1. Figure 2 and results CRISPRi results section – We have clarified the CRISPR-interference schematic and figure labels (Figure 2C and 2D) to make it clearer that EF-P is being depleted in the various backgrounds. We have edited the results section to go with the order of results presented in the figure.
2. We have clarified that the binding site of YebC2 on the ribosome is unknown in the discussion.
3. We describe the ribosome association discussion according to the helpful suggestions of reviewer 1.

Reviewers' comments:

Reviewer's Responses to Questions
Reviewer #1: Hong and colleagues characterise a novel translation factor, YevbC2, and show that the factor is involved in resolving ribosomal stalls on polyproline tracts. The results are interesting and convincing, although somewhat limited in mechanistic detail. However, as this study is one of the first reports describing the biological function of the factor, the level of detail is adequate. I have several comments / suggestions.

We thank the reviewer for their helpful suggestions which have significantly improved the manuscript.

Throughout the text, including figure legends, the authors refer to 'severe growth and fitness defects'. 'Severe' is too strong a word here, I feel. I would use something less dramatic.

We have edited 'severe' to 'significant'

Line 58: 'The Escherichia coli homolog of YfmR, Uup'. I think here one could be more specific and say 'orthologue' – while Uup is, indeed, a homologue of YfmR, so are the other ABCF ATPases. However, the connection between Uup and YfmR is tighter than that.

These are good points, we have changed homolog to orthologue.

YebC2 over-expression rescues the synthetic fitness defect of ΔefpΔyfmR: could you please also show serial dilutions data in addition to colony size measurements? Or growth rate measurements? colony size measurements are a rather exotic way to assess fitness.

We have added growth measurements (Figure 2A and Figure S1).

Have the authors attempted generating a ΔefpΔyebC2 double KO strain?

I assume this is a typo and the reviewer means ΔefpΔyebC double KO. We constructed this strain and have added data showing that it does not have increased ribosome stalling on the polyproline reporter (Figure 4B).

Line 189: 'YebC2 associates with 70S ribosomes'. Polysome analysis shows that YebC2 signal is present in 30S, 50S and 70S fractions, but not in polysomes. I would be careful and not make strong concussions about 70S being the target. 30S and 50S signal is similarly strong. Moreover, there is no signal on the polysomes… Obviously, the factor might not interact stably with the target ribosomal complex and be lost during the centrifugation. Please reword 'We found that YebC2 co-migrates with ribosomes and was strongly associated with 70S ribosomes' and highlight the lack of the interaction with polysomes.

We have edited this section to say "We did not detect YebC2 association with polysomes, suggesting that either YebC2 does not interact with the most actively translating ribosomes or that the interaction is transient." Line 175.

Figure 4: while bootstraps are mentioned in the text, I somehow miss them on the figure / figure legend. Please revise for clarity so they are more obvious. I would compare the structures (experimental or AF2-generated) for YebC2 and YebC: what is the structural difference? What makes one a translation factor and the other a transcription factor?

We have bolded the bootstrap value and added it to the figure legend. We have added AF-generated models of YebC2 and YebC overlayed with one another. In figure 5A and B we show potentially important differences in electrostatics at the surfaces of these proteins.

Figure 5: please italicise yebC2 gene.

Corrected.

Line 277, 'EfpLhave'. A space is missing.

Corrected.

Line 289: 'Helix 89' – please specify if it is a small or a large subunit, and where on the subunit the helix is located. Not everybody is familiar with ribosomal landmarks.

We have elaborated on our description of Helix 89. Please also see the model of sites found by Ignatov et al to crosslink with yebC2 in our response to Reveiewer 2 below. (Line 271-274)

Line 49 and 298: 'P-site' should be P site. The hyphen would be needed when we have a compound adjective, like 'P-site tRNA', line 306. When we are referring to the P site as such, no hyphen is needed. Same applies to line 287, 290, 309 'A-site'.

Corrected.

Line 33, ‘E.coli’. A space is missing.

Corrected.

Reviewer #2: The manuscript by Hong et al, describes the role of YebC2 (YeeI) in preventing ribosome stalling in Bacillus subtilis. Using clever and clear genetics they show that depletion of YebC2 impairs viability of cells lacking Efp and that the absence of YebC2 increases the rate of ribosome stalling and abortive translation. Finally, they show that YebC2 associates with ribosomes and promotes 70S ribosome and polysome production. They round out the characterization with nice phylogenetic analyses. The work is interesting and sound. I only have a few minor comments.

We thank the reviewer for their helpful suggestions which have significantly improved the manuscript.

Line 124. The ymfR yebC2 double mutant comes a bit out of nowhere here. What is the phenotype of the ymfR yebC2 double? Maybe just start with depleting efp in the single mutants and describe relative fitness before moving on to the double. Starting the paragraph with the undescribed double creates a massive leap in logic that was distracting/confusing. I note that what I described above is also consistent with the order of strains in figure 2A.

We agree our original wording was quite clunky, we have edited to go with the order of strains in Figure 2D.

Line 166. What is known about truncated peptide production in B. subtilis? Is this like the ssrA trans-translation system? And why is the truncated product not destroyed by proteolysis? In general, this statement needs better support/explanation.

In *B. subtilis*, ribosomes that stall mid message are removed from the mRNA by a splitting factor called MutS2 (Park 2024). The large subunit remains obstructed by the peptidyl-tRNA and RqcH then adds alanines to the peptide to expose the peptidyl-tRNA bond to the cytosol and PTH then cleaves the bond between the peptide and tRNA. The alanine tag targets the peptide for degradation.

Our observation of the stability of truncated peptide produced from stalled ribosomes in *B. subtilis* has also been reported by others (see Takada NAR 2024 and Park EMBO 2024 Fig 3D with ApdA staller). We have also puzzled over the unexpected stability of stalled peptides in *B subtilis*. We suspect two possibilities which are 1) the RqcH tagging system is not as efficient as *trans*-translation and so the peptide hangs around longer and/or 2) truncated peptides ending in consecutive prolines are not good substrates for RqcH and are therefore not as efficiently alanine tagged.

We note that in Δ*efp*Δ*yebC2* cells and in Δ*efp*Δ*yfmR* cells there is a detectable decrease in full-length peptide along with the increase in the stalled peptide. And, in both cases, over-expressing

YebC2 causes an increase in full-length protein and not just a decrease in truncated protein. These results suggest that YebC2 promotes translation rather than only degradation of the truncated peptide. However, since the degradation of truncated peptide in B. subtilis depends on alanine-tagging, it is certainly possible that YebC2 (and EF-P and YfmR) also promote peptide bond formation in the context of RqcH alanine-tagging. We have added some discussion of these possibilities to the discussion section. (Lines 276-287.)

Figure 4 and results. What distinguishes YebC from YebC2? Are there particular domains added/missing? Does YebC2 lack the HTH or whatever promotes DNA binding of the YebC subfamily? A multiple sequence alignment comparing YebC to YebC2 proteins in prominent model organisms might be useful to see at a glance how/where the subfamilies differ.

While there were no obvious differences between YebC and YebC2 in protein alignment or in AlphaFold models, we found that YebC and YebC2 have different roles *in vivo*. While YebC2 can reduce levels of stalled peptide in Δ*efp* and Δ*efp*Δ*yebC2* backgrounds, YebC (YrbC) expression had no effect.

Motivated by the reviewer's suggestion we modeled electrostatics of these proteins and were able to identify potential differences in a helix in Domain I on the surface of the proteins (Fig. 5A and 5B). To test whether these differences are important for YebC2's function we swapped Domain I between YebC2 and YrbC. When we expressed YebC2 encoding Domain I from YrbC ($\text{YebC}^{\text{YrbC-D1}}$), this chimeric version of the protein failed to complement the ribosome stalling phenotype of Δ*efp*Δ*yebC2*. However, when we expressed YrbC encoding Domain I of YebC2 ($\text{YrbC}^{\text{YebC2-D1}}$), this chimeric version complemented the Δ*efp*Δ*yebC2* ribosome stalling phenotype to the same level as wild-type YebC2. We have added this data in Fig 5D.

Phylogenetic distribution. Any chance that the presence of YebC2 has anything to do with rapid growth rates?

This is a great question. I don't feel confident assigning conclusions based on growth rate, but it is interesting that many of the species that have YebC2 are related to *E. coli* and *B. subtilis*. Maybe laboratory adaptation selects for faster growth rate and this could be a selective pressure for YebC2? On the other hand, we observed more pronounced rescue of Δ*efp*Δ*yfmR* by YebC2 over-expression at 30˚C than at 37˚C. That result suggests that YebC2 may be more important at the slower growth rate of 30˚C, or that there is more translation stress at lower temperatures.

Line 289. What is Helix 89?

We have elaborated our description of Helix 89 in the discussion section and have generated a model here for the reviewer in our response because these data are very interesting.

Helix 89 is a helix in the 23S rRNA that extends from the peptidyl-transferase center along the A-site. The residues predicted in the pre-print by Ignatov et al to interact with YebC2 are at the base of the helix close to the PTC (we have colored these RNA bases of H89 that YebC2 cross-

linked in magenta). The residues are extremely close to where the P- and A-site tRNAs come together for peptidyl transfer.

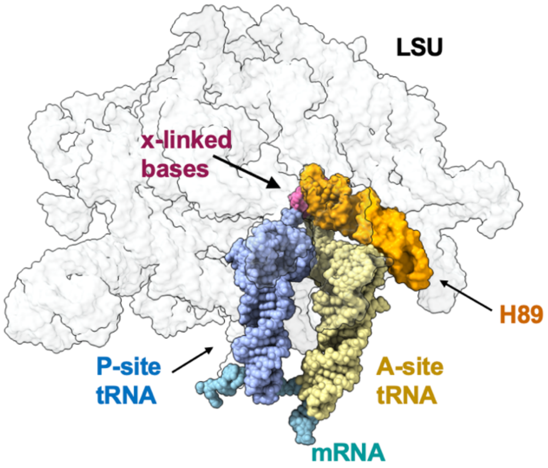

Reviewer #3: This manuscript describes the identification of YebC2 as a translation factor of B. subtilis that resolves ribosome stalling at poly-Pro sequences. The overall conclusion is not that novel, because (1) the homologs of the protein in human and in S. pyogenes have been demonstrated with such role, and (2) the protein was identified from the same screen that the authors published previously for proteins that can rescue the efp-KO defect, knowing that efp is the main factor that resolves ribosome stalling at poly-Pro sequences. The lack of novelty of the overall conclusion is compounded by the incomplete analysis of the YebC2 protein in multiple places, where the authors simply stated that "further experiments are necessary". In fact, these are exactly the experiments that are necessary to convincingly demonstrate the proposed role of YebC2. Below both major and minor weaknesses of the manuscript are listed.

We thank the reviewer for their helpful comments and suggestions which greatly improve the manuscript.

Our work adds novel data not previously reported. Most notably the physiological importance of YebC2 in the absence of EF-P and YfmR and the bioinformatic analysis of the YebC2 family which adds a clearer picture of the evolutionary distinction of the YebC2 family of translation factors. We have also determined domains of YebC2 which are important for its function. These contributions are important for helping to illustrate a more complete picture of YebC2 function in bacteria.

We also note that our work is highly complementary and supportive of the outstanding work of Brischigliaro et al. and Ignatov et al. The immediate support of those two recent papers using different approaches and adding additional findings is beneficial to the community of labs which are working in this important area.

Conceptual weaknesses:
1. The authors stated that YebC2 is “independent” of EF-P and YfmR as a factor that resolves ribosome stalling at poly-Pro sequences. However, “independent” needs to be quantitatively demonstrated by measuring the effect of each protein. If EF-P-KO has a growth defect of x-fold, YfmR-KO has an effect of y-fold, and YebC2-KO has an effect of z-fold, these fold changes need to be directly compared. It is a mis-opportunity that the data in Figure 2B is not more carefully analyzed.

Single knockouts of Δ*efp*, Δ*yfmR*, or Δ*yebC2* do not exhibit a growth defect on their own (Fig. 2A and 2B and 2D). This is expected, since deletion of genes that have redundant function typically does not result in a strong phenotype until more than one is deleted. Since EF-P is the main protein that resolves stalling at polyprolines it is expected that ΔyebC2 or Δ*yfmR* only exhibit a strong phenotype when EF-P is absent. This is exactly what we observe and report in Figure 2.

Even if Yeb2C has a rescue effect in the strain of EF-P-KO/YfmR-KO, this does not mean that Yeb2DC is independent of the two other genes. Until further analysis, the claim of an ”independent” effect is not justified.

The ability of YebC2 to rescue cellular fitness in the absence of EF-P and YfmR does indeed indicate that it is functional for supporting growth independent of the help of EF-P and YfmR. If YebC2 were dependent on EF-P or YfmR for its activity, then one of these genes would need to be present for it to be able to support growth. Since that is not the case, we conclude that YebC2 retains rescue function that is independent of these two proteins.

As further due diligence to satisfy the reviewer’s concern, we have performed an experiment with the ribosome stalling reporter to show that over-expression of YebC2 in the Δ*efp*Δ*yfmR* background reduces the proportion of the stalled peptide (Fig 3A). Again, if YebC2 needed EF-P or YfmR to reduce ribosome stalling then it would not be able to reduce ribosome stalling in the absence of these two factors. Since it reduces stalling in the absence of these two factors this is sufficient evidence that it can function on its own.

2. Just because YebC2 is associated with the 70S, it does not mean that it facilitates translation of the ribosome. It may provide a quality control that regulates the ribosome activity. Additionally, it appears that YebC2-KO leads to accumulation of 50S and 30S, indicating that it prevents the 70S assembly. An explanation of this role is completely absent from the manuscript.

We thank the reviewer for raising these interesting points and have added a paragraph to the discussion to describe this possibility (Lines 277-288). The accumulation of 50S and 30S is also observed for Δ*efp*Δ*yfmR* knockouts (Hong et al 2024 and Takada et al 2024) and we have added some text to reference this observation (Line 102-104). The reason for accumulation of 30S and 50S ribosomes is likely either the slower growth rate of the cells or the failure to produce FMT which has a polyproline stall near the 5’ end of the gene. These possibilities were discussed in Hong et al 2024.

3. While phylogenetic analysis showed that YebC2 is separated from other members of the YebC family, this does not mean it has only a function in translation and not in transcription as in other members of the YebC family. The example is the E. coli YebC2 enzyme, which appears to have a role in both. The conclusion made by the authors is again very superficial and without experimental backing.

We thank the reviewer for this suggestion and have added an extensive exploration of the role of the YebC paralog and identified domains in YebC2 and YebC which account for differences between the two proteins (Fig 4 and 5). In *B. subtilis* the YebC protein is called YrbC. We show that over-expression of YrbC does not reduce ribosome stalling on a polyproline tract. We also show that Δ*efp*Δ*yrbC* cells do not exhibit increased ribosome stalling at polyprolines. We also modeled the structure and electrostatics of these proteins and identified oppositely charged residues on the surfaces of these proteins in Domain I. YrbC encodes a negatively charged region on the surface of Domain I while YebC2 encodes a positively charged surface at the same region. When we swapped this domain between the two proteins we were able to see that this domain is important for YebC2's activity. We found that YrbC gained YebC2's function if provided with YebC2's Domain I.

Technical weaknesses:
1. The authors used the word "more essential" multiple times in the text. In Genetics, "essential" is a definitive word – either it is required or not required for growth. The phrase "more essential" is unclear.

We have corrected "more essential" to "more important for fitness" to clarify

2. Table 1 can go to the Supplement.

We have removed Table 1 and just described in words.

3. Figure 2A, the Y axis is unclear. It should be #generations per hour.

We have changed to doublings per hour, consistent with prokaryotic nomenclature.

4. The writing in line 130 is very difficult to read. The authors wanted to say "when compared to when EF-P was knocked out alone"?

We have re-written this results section and updated our CRISPR depletion schematic to clarify this experiment.

5. The data in Figure 2A of the spot test do not convey the results. The most critical presentation should be the effect of EF-P-KO. This figure should be re-done.

We have re-done this figure adding an EF-P KO control. We hope the reviewer also notes that EF-P is the protein that is being depleted using CRISPR interference. We have improved our

schematic and labeling of the figure to make it more clear that EF-P is what is being depleted since this is indeed the critical aspect of the finding presented in the figure.

6. In contrast to authors' claim, there is no effect of yebC2 complementation at 37 oC in Figure 2B. The authors incorrectly interpreted the results?

Although the bars look similar in Figure 2B at 37˚C the results of the unpaired T-test with Welch's correction indicate significant rescue.

As further due diligence we have performed an additional experiment and show that there is significant rescue in Δ*efp*Δ*yfmR* with YebC2 over-expression in liquid culture at 37˚C (Fig S1). The rescue in liquid culture is even more pronounced.

7. Line 174. The "additive increase in ribosome stalling for the .....", the word "additive" needs to be justified. Without a quantitative measurement, this word was incorrectly used.

We have edited this line since we note that the increase in ribosome stalling in Δ*efp*Δ*yebC2* cells exhibits a synergistic, rather than simply additive, increase than either of the single deletions. This is expected since EF-P and YebC2 likely have overlapping targets. When either YebC2 or EF-P is present on its own it may be able to handle a particular set of stalled ribosomes, but when both proteins are absent that set of stalled ribosomes is no longer rescued and would result in the synergistic increase we observe.

8. Figure 3A needs a control of the reporter gene, not a poly-Pro sequence. In the same figure, "stalled" is mis-leading. Instead, "truncated peptide" would be more appropriate.

The advantage of this type of reporter is that the full-length protein serves as an internal control to which levels of stalled protein can be directly compared. Reporters similar to this one are very standard in the field and have been widely used in Hong et al 2024, Takada et al 2024, and in Ignatov et al. 2024.

"Stalled" is the nomenclature used by others in the field and conveys the meaning better than truncated since ribosome stalling is what leads to the truncated peptide.

9. Figure 3B, where does YebC2 bind to the ribosome? It appears to bind 70S, 50S, and 30S. What is the meaning of this binding? In the same figure, a control of EF-P is necessary, but lacking.

It is typical that proteins become disassociated from their partners while running through a high percentage of sucrose for 3 hours (we are in agreement with reviewer 1 with respect to this). The same phenomenon can be seen for EF-Tu, our positive control for ribosome association. We have revised our wording in both the results and discussion to discuss apparent co-migration of YebC2 with other ribosomal subunits. EF-P is not an informative control in this experiment because EF-P associates very transiently with the ribosome. We and others have not found a strong association of EF-P with ribosomes on sucrose density gradients due to the highly transient nature of EF-P's interaction.

10. Evidence that YebC2 binding to the A site should be directly demonstrated. This is lacking and thus the proposed model is incomplete.

The precise location of YebC2 binding will require a structure which is beyond the scope of this manuscript. The potential for A site binding was a speculative portion of the discussion in the original manuscript. In the revised manuscript we have removed this speculation.

11. A simple prediction of YebC2 by an Alpha-Fold modeling should have been done to predict its structure. This is lacking, making the proposed model incomplete.

We have added AlphaFold models of both YebC and YebC2 from *Bacillus subtilis* (Fig. 5). Based on the reviewer's suggestion we also further modeled electrostatics of these proteins and were able to identify potential differences in a helix in Domain I on the surface of the proteins (Fig. 5). These residues are in Domain I. To test whether these differences are important for YebC2's function we swapped Domain I between YebC2 and YrbC. When we expressed YebC2 encoding Domain I from YrbC ($\text{YebC}^{\text{YrbC-D1}}$), this chimeric version of the protein failed to complement the ribosome stalling phenotype of Δ*efp*Δ*yebC2*. However, when we expressed YrbC encoding Domain I of YebC2 ($\text{YrbC}^{\text{YebC2-D1}}$), this chimeric version complemented the Δ*efp*Δ*yebC2* ribosome stalling phenotype to the same level as wild-type YebC2. We have added this data in Fig 4. We thank the reviewer for this suggestion.
